# The burden of chronic obstructive pulmonary disease in open heart surgery—a retrospective cohort analysis of postoperative complications: STROBE compliant

**DOI:** 10.1097/MD.0000000000019675

**Published:** 2020-03-27

**Authors:** Aleksandra Szylińska, Katarzyna Kotfis, Mariusz Listewnik, Mirosław Brykczyński, Annachiara Marra, Iwona Rotter

**Affiliations:** a Department of Medical Rehabilitation and Clinical Physiotherapy, Pomeranian Medical University, ul. Żołnierska 54; b Department of Anesthesiology, Intensive Therapy and Acute Intoxications, Pomeranian Medical University; c Department of Cardiac Surgery, Pomeranian Medical University, al. Powstańców Wlkp. 72, Szczecin, Poland; d Department of Neurosciences, Reproductive and Odontostomatological Sciences, University of Naples, Federico II, Naples, Italy.

**Keywords:** cardiac surgery, chronic obstructive pulmonary disease, delirium, mortality, pulmonary complications

## Abstract

Chronic obstructive pulmonary disease (COPD) has a major impact on mortality and morbidity in patients undergoing cardiac surgery. Mortality risk increases by 50% in patients who were re-intubated or required prolonged mechanical ventilation after the operation. The aim of this study was to assess the impact of COPD on the prediction of postoperative complications and outcome including intensive care unit (ICU) and hospital stay, postoperative morbidity and mortality in patients undergoing all types of cardiac surgery.

We performed a retrospective cohort analysis of prospectively collected data from a tertiary cardiac surgery department of a university hospital between 2014 and 2016. We divided patients undergoing cardiac surgery into 2 sub-groups – the first – with a clinical diagnosis of COPD (n = 198) and the second comprised all other non-COPD patients (n = 2980).

Among patients with COPD a longer intubation time (*P* = .039), longer ICU stay (*P* < .001) and longer hospitalization time (*P* = .006) was noted as compared with non-COPD patients. Patients with COPD required reintubation more often than non-COPD patients, reintubation occurring twice, 19 (9.60%) versus 144 (4.83%) *P* = .002, reintubation occurring 3 or more times, 7 (3.54%) versus 34 (1.14%) *P* = .006. Mortality within 30 days after surgery was higher in patients with pulmonary problems before surgery (*P* = .003). Multivariable logistic regression analysis corrected for interfering variables showed an increased risk of postoperative bronchoconstriction (odds ratio [OR] = 4.40, *P* = .002), respiratory failure (OR = 1.67, *P* = .018), atrial fibrillation (OR = 1.45, *P* = .023), and use of hemofiltration (OR = 1.60, *P* = .029) for patients with COPD.

Patients with COPD undergoing all types of cardiac surgery are at increased risk of respiratory complications and mortality. The occurrence of COPD was associated with longer ICU and hospital stay. In COPD patients, undergoing cardiac surgery, treatment strategies aimed at preventing reintubation and early weaning mechanical ventilation must be employed to reduce postoperative complications.

## 1 Introduction

Chronic obstructive pulmonary disease (COPD) is a systemic disease with various co-morbid conditions, including cardiovascular disease, depression, chronic kidney disease, and osteoporosis.^[1–6]^ Epidemiological studies have reported that the prevalence of COPD in the general population ranges from 8 to 20%, and the world population of COPD patients reached 7.3 billion cases in 2015.^[7,8]^ People with COPD have a higher risk of cardiovascular diseases, most commonly coronary artery disease, atrial fibrillation, myocardial infarction (MI) and heart failure, but also present with poorer long-term survival and outcomes after MI.^[9,10]^

Due to advancement in perioperative care and improvement in anesthetic and surgical techniques, an increasing number of COPD patients are qualified for open-heart procedures and accept this treatment option despite increased risk, as compared with non-COPD patients.

To stratify perioperative risk in cardiac surgery, the European System for Cardiac Operative Risk Evaluation (EuroSCORE) system, which is widely used, includes chronic pulmonary disease as an independent prognostic factor for surgical mortality.^[11]^ Postoperative complications, such as respiratory failure, requiring repeated intubation or prolonged support of mechanical ventilation, are common in COPD patients undergoing coronary artery bypass grafting (CABG) procedure with the use of cardiopulmonary by-pass.^[12,13]^ Many patients with COPD undergoing CABG are at higher risk for postoperative complications, including pneumonia, respiratory failure, stroke, wound infection, renal failure and require prolonged stay in the intensive care unit (ICU) and in the hospital after surgery.^[12,14]^ Yet majority of the studies that report data regarding COPD patients concentrate on those undergoing CABG and very few have reported outcome in COPD patients undergoing other kinds of cardiac surgery.

The aim of this study was to assess the impact of COPD on the prediction of postoperative complications and outcome including ICU stay, hospital stay, postoperative complications, and mortality in patients undergoing all kinds of cardiac surgery requiring general anesthesia in 1 cardiac surgery center.

## 2 Material and methods

### 2.1 Patients population

Based on data collected from January 1st, 2014 to December 31st, 2016, a retrospective cohort analysis was performed at the Department of Cardiac Surgery, Pomeranian Medical University in Szczecin. The study involved 3467 patients qualified for cardiac surgery. The study excluded patients undergoing minimally invasive procedures, without the use of general anesthesia and patients without complete information regarding the perioperative surgery.

### 2.2 Data analysis

Pre-surgery medical records provided information on demographic data (age, sex, body mass index), comorbidities, and previous history of hypertension, diabetes, atherosclerosis, angina pain, MI, atrial fibrillation, heart failure, cancer, neurological diseases, kidney disease, and COPD. Routine preoperative, perioperative, and postoperative examinations yielded data on baseline ejection fraction, based on echocardiography, C-reactive protein, creatinine and glomerular filtration rate, phosphocreatine kinase, and glycated hemoglobin, aortic clamping time, perfusion time and reperfusion and intubation time. The operational risk assessment was performed using the EuroScore II (ESL) scale. After surgery, information about 30-day mortality, postoperative complications (stroke, transient ischemic attack, convulsions, delirium, cardiogenic shock, pneumothorax, respiratory failure, acute kidney injury, atrial fibrillation), ICU length of stay and hospital length of stay at the Cardiac Surgery Department were obtained.

**Figure 1:**
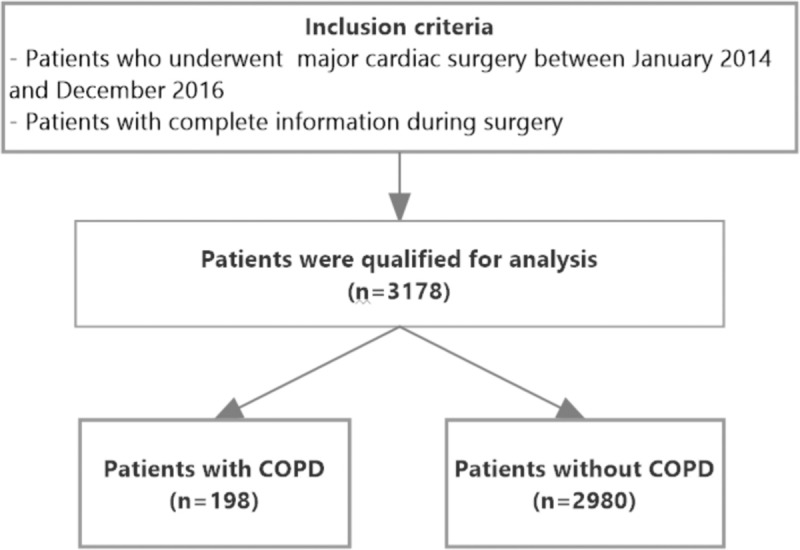
Study flowchart. COPD = chronic obstructive pulmonary disease, n = number of patients.

We included all consecutive patients undergoing cardiac surgery between 2014 and 2016 and divided them into subgroups depending on the diagnosis of COPD. The first group included patients with a clinical diagnosis of COPD according to the GOLD criteria stages I to IV.^[5]^ The second group included patients without a clinical diagnosis of COPD. Figure 1 presents study flowchart showing division into subgroups.

### 2.3 Ethics

Due to the retrospective nature of the study design, the study received a waiver from the Bioethical Committee of the Pomeranian Medical University in Szczecin, Poland, decision no (KB-0012/277/10/18). Nevertheless, informed consent was obtained before surgery from each patient for data collection. The study was performed in accordance with the Declaration of Helsinki and STROBE guidelines for epidemiological studies.

### 2.4 Statistical analysis

To evaluate the normality of the distribution of the studied variables we used the Shapiro-Wilk test. Descriptive statistics for the 2 groups (COPD and non-COPD) were made for quantitative data using the Mann–Whitney *U* test, the results were presented using mean and standard deviation. We performed, analysis of qualitative data using the Chi-square test or Chi-square test with Yates correction for the 2 groups, the data were presented using the number of patients and percentages. The odds ratio (OR) of postoperative complications in COPD patients was obtained through the use of logistic regression. Univariable logistic regression and multiple logistic regression adjusted by age, sex, body mass index, smoking, and baseline medical comorbidities were performed. The results of regression were presented with the value of the OR with 95% confidence intervals and the statistical significance value. All data were analyzed using licensed software Statistica 12 (StatSoft, Inc, Tulsa, OK). *P*-value of <.05 was regarded as statistically significant.

## 3 Results

### 3.1 Baseline characteristics

We analyzed 3178 patients, 198 (6.23%) with COPD and 2980 (93.77%) without COPD. One of the elements of the ESL scale is chronic lung disease, therefore patients with COPD presented a much higher operating risk scale (4.76 ± 6.79) compared to patients without COPD (3.15 ± 4.66), *P* < .001. Table 1 presents demographic data, basic comorbidities and preoperative and intraoperative parameters divided by the occurrence of COPD. The average age of patients not suffering from COPD was 65.7 ± 9.7 years, while those suffering from COPD were significantly older 68.6 ± 8.1 years (*P* < .001). Among patients with COPD, more people smoked cigarettes (*P* = .002) and the length of cigarette smoking was significantly higher (*P* < .001). Of the preoperative comorbidities, statistically significant differences between the groups were obtained only regarding transient ischemic attacks (*P* = .007), congestive heart failure New York Heart Association III and IV (*P* = .002) and persistent atrial fibrillation (*P* = .005). A larger number of COPD patients also suffered from pulmonary hypertension (*P* = .043). Patients with COPD on the day of admission to the cardiac surgery department had a significantly lower level of glomerular filtration rate than patients without COPD (*P* = .007). The use of hemofiltration during surgery was more often reported in patients with COPD (*P* = .032).

**Table 1:**
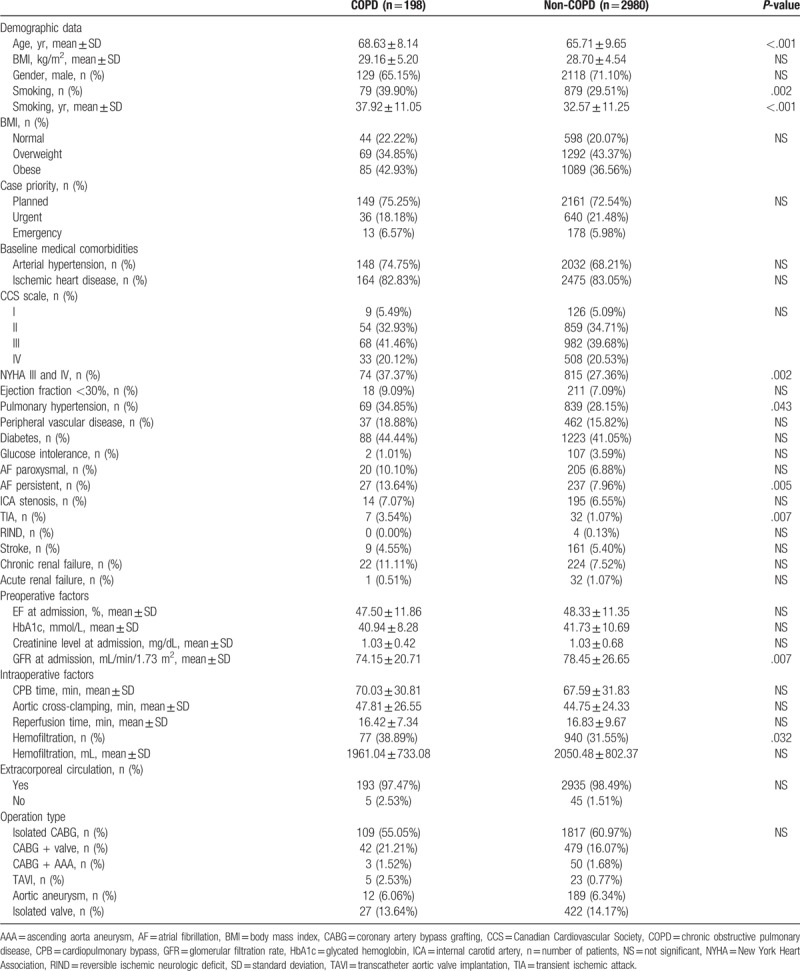
Comparison of baseline demographics and characteristics for study groups.

### 3.2 Postoperative outcome and complications

Table 2 presents a comparison of postoperative parameters and the occurrence of postoperative complications between patients undergoing cardiac surgery with the co-accompanying COPD and without COPD. After surgery, a significantly higher level of creatinine was measured in patients with COPD than in those without pulmonary problems (*P* < .001). Among patients with obstructive pulmonary disease, a significantly longer intubation time (*P* = .039), ICU stay (*P* < .001), and hospitalization (*P* = .006) were observed compared to the remaining patients. Attention is drawn to the fact that patients with COPD had a significantly higher number of episodes of bronchospasm after surgery (*P* = .001) and respiratory failure (*P* = .001). These patients were also more often re-intubated compared to people without COPD, 2 intubations were required in 19 (9.60%) versus 144 (4.83%) patients (*P* = .002), while 3 and more intubations were required in 7 (3.54%) versus 34 (1.14%) patients (*P* = .006). Mortality within 30 days after surgery was higher in patients with pulmonary problems before surgery (*P* = .003). Interestingly, although the number of episodes of pneumonia was higher in COPD patients, this was not statistically significant - 11 (5.56%) versus 96 (3.22%) (*P* = .119).

**Table 2:**
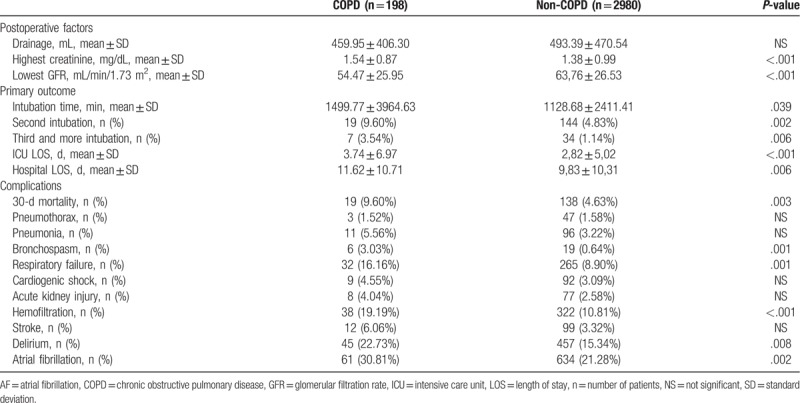
Comparison postoperative outcome and complications for patients with COPD and patients without COPD.

The results of univariable logistic regression (Table 3) confirm the association of COPD with the occurrence of increased mortality up to 30 days after surgery (OR = 2.186, *P* = .002). The risk of respiratory failure was twice as high for COPD than for non-COPD patients (OR = 1.975, *P* = .001). In addition, COPD patients had an increased risk of bronchospasm (OR = 4.868, *P* = .001), stroke (OR = 1.877, *P* = .045), atrial fibrillation (OR = 1.648, *P* = .002), and delirium (OR = 1.642, *P* = .006). Among patients with COPD, there was a slight but increased risk of a longer ICU (OR = 1.022, *P* = .022) and hospital length of stay (OR = 1.011, *P* = .028).

**Table 3:**
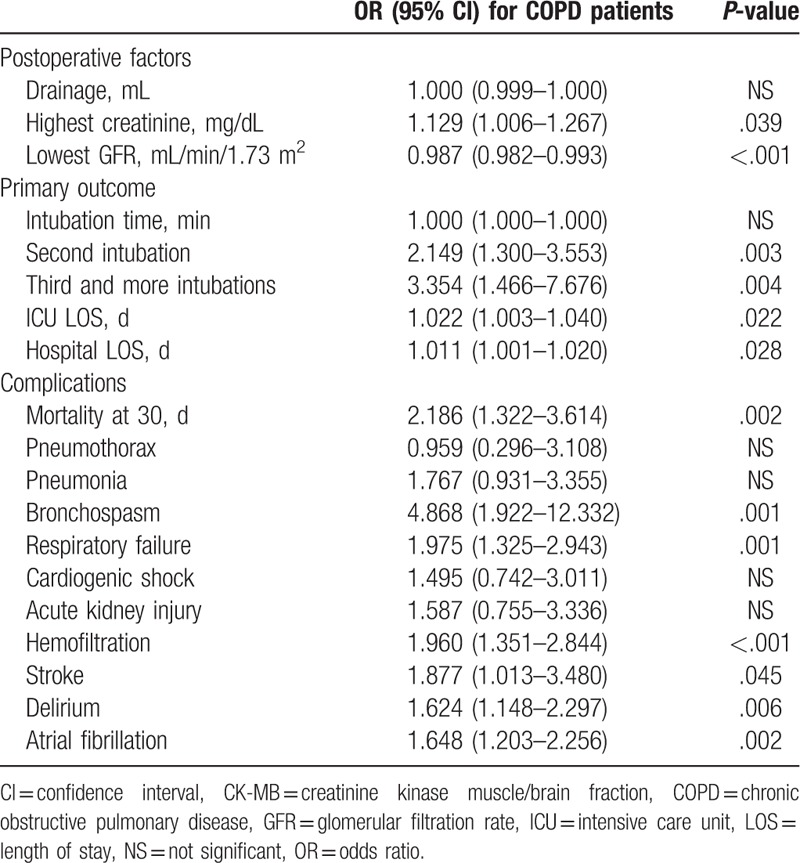
Univariable logistic regression analysis for patients with COPD.

The assessment of the relationship between COPD and the occurrence of postoperative complications is presented in Table 4. The 30-day mortality rate and postoperative adverse events were presented using multivariable OR adjusted to selected demographic factors and comorbidities. After correcting the interfering variables, an independent association of COPD was obtained in patients after cardiac surgery with postoperative complications. There was an increased risk of postoperative bronchoconstriction (OR = 4.40, *P* = .002), respiratory failure (OR = 1.67, *P* = .018), atrial fibrillation (OR = 1.45, *P* = .023), and the use of hemofiltration (OR = 1.60, *P* = .029).

**Table 4:**
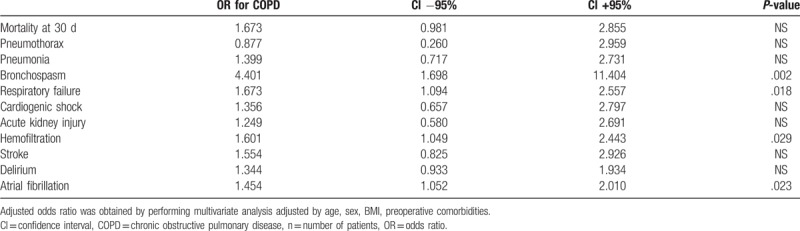
Multivariable logistic regression analysis for patients with COPD.

## 4 Discussion

In this retrospective cohort analysis of prospectively collected involving patients undergoing cardiac surgery with a clinical diagnosis of COPD, we found that patients with preoperative COPD are more often exposed to reintubation and the occurrence of respiratory complications after all types of cardiac surgery, which results in a longer ICU and hospital length of stay. Our study has shown that among patients with COPD, bronchoconstriction occurred 4 times more often, and respiratory failure was twice as likely compared to patients without COPD.

In a prospective study of patients after CABG, Woods et al showed an increased number of pulmonary complications (prolonged ventilation, pulmonary embolism, and pneumonia) in patients with COPD.^[15]^ Similar results were obtained Saleh et al, presented a retrospective analysis of patients undergoing isolated CABG. Among patients with moderate COPD, a significantly longer duration of intubation (OR = 1.60, *P* = .003) was showed; however, patients with severe COPD had more than 2 times the risk of prolonged intubation.^[16]^ Similarly, in a group of patients with COPD (diagnosis based on GOLD or LLN criteria) after cardiac surgery, McKeon et al demonstrated a longer intubation period, a longer ICU and hospital length of stay as compared to the control group without COPD.^[17]^ Cani et al examined 2 groups of patients after coronary artery bypass grafts with pulmonary complications and without pulmonary complications and showed a much longer hospital stay in patients with pulmonary complications.^[18]^ Efird et al showed that prolonged length of stay after CABG was an independent predictor of decreased long-term survival (adjusted hazard ratio [HR] 6.0 [4.4–8.2]; *P* trend < .001).^[14]^

According to the Society of Chest Surgeons and the European Heart Operational Risk Assessment System (EuroSCORE) in patients undergoing cardiac surgery, COPD is an independent predictor of hospital mortality.^[19,20,22–26]^ Our results showed more than a 2-fold increase in mortality up to 30 days after cardiac surgery of patients with COPD (OR = 2.186, *P* = .002). Huang et al carried out a randomized multi-center left main coronary artery disease studies of patients undergoing cardiac revascularization. Regardless of, whether percutaneous coronary intervention (PCI) or CABG was performed, COPD was independently associated with an increase in 30-day and 3-year mortality rates compared to patients without COPD.^[19]^ Similar results were obtained by Lin et al who performed a retrospective analysis between 2000 and 2012 of 215,275 patients after PCI. In patients with COPD, the analysis showed an increased risk of hospital mortality (OR = 1.83, *P* < .001).^[20]^ Other results also showed an increase in mortality in patients after PCI (HR = 2.45, *P* < .0001).^[21]^ Among patients undergoing surgery, CABG Ried et al showed an increase in 30-day mortality.^[22]^

Saleh et al demonstrated in patients with CABG an increase in early mortality in patients with mild (1.4%), moderate (2.9%) and severe COPD (5.7%) (*P* < .001).^[16]^

O’Boyle et al, in an analysis of 13,337 primary CABG procedures with a median follow-up of 7 years, demonstrated that all types of COPD (obstructive, restrictive, or mixed) are significant factors determining long-term survival.^[27]^ Angouras et al reported a HR of 1.28 (95% confidence interval, 1.11–1.47, *P* = .001) for long-term mortality (mean follow-up 7.6 years).^[28]^

Our results showed also a much higher need for hemofiltration (OR adjusted = 1.60, *P* = .029) in patients with COPD after cardiac surgery than in patients without COPD. A similar result was obtained by Chen et al that has shown an independent relationship between the occurrence of COPD and kidney disease (HR adjusted = 1.61, *P* < .001).^[29]^ This necessity for hemofiltration in COPD patients observed in our study may be explained by relative pulmonary fluid overload that does not always indicate acute kidney injury.

## 5 Limitations

This was a retrospective single-center study, and the results may not be generalizable to other cardiac surgery centers, yet the majority of studies regarding COPD patients are single-center reports, therefore there should be a call for multi-center epidemiological studies, including all types of cardiac surgery, not only CABG. Moreover, our follow-up was performed early, up to 30 days postoperatively and a longer follow-up period would add more insight into the long-term mortality in COPD patients. Another limitation of our research was the lack of spirometric testing result which made impossible to assess the severity of COPD. Although we have reported data regarding delirium in this group of patients with COPD, we have not reported the intensity of pain. Both delirium and pain associated with prolonged intubation time should be reported in COPD patients and can be easily assessed using dedicated scales, even in non-verbal critically ill patients.^[30–32]^

## 6 Conclusions

Patients with COPD undergoing all types of cardiac surgery are at increased risk of respiratory complications, mortality, and longer ICU and hospital length of stay. In COPD patients undergoing cardiac surgery treatment strategies aimed at preventing reintubation and early weaning from mechanical ventilation must be employed to reduce postoperative complications including the length of ICU and hospital stay, as well as mortality.

## Acknowledgments

The authors would like to thank the employees of the Cardiac Surgery Department of the Pomeranian Medical University in Szczecin, Poland.

## Author contributions

**Conceptualization:** Aleksandra Szylińska, Katarzyna Kotfis, Mariusz Listewnik, Iwona Rotter.

**Data curation:** Aleksandra Szylińska, Katarzyna Kotfis, Mariusz Listewnik.

**Formal analysis:** Aleksandra Szylińska, Katarzyna Kotfis, Annachiara Marra.

**Investigation:** Katarzyna Kotfis, Annachiara Marra.

**Methodology:** Aleksandra Szylińska.

**Project administration:** Aleksandra Szylińska, Mirosław Brykczyński, Iwona Rotter.

**Resources:** Aleksandra Szylińska, Mariusz Listewnik.

**Supervision:** Katarzyna Kotfis, Mariusz Listewnik, Mirosław Brykczyński, Annachiara Marra, Iwona Rotter.

**Validation:** Katarzyna Kotfis, Mariusz Listewnik, Mirosław Brykczyński, Annachiara Marra, Iwona Rotter.

**Writing – original draft:** Aleksandra Szylińska, Katarzyna Kotfis, Mariusz Listewnik, Mirosław Brykczyński, Annachiara Marra, Iwona Rotter.

## References

[1] Barnes PJ Celli BR Systemic manifestations and comorbidities of COPD. Eur Respir J 2009;33:1165–85. 19407051 10.1183/09031936.00128008

[2] Incalzi RA Corsonello A Pedone C Chronic renal failure: a neglected comorbidity of COPD. Chest 2010;137:831–7. 19903974 10.1378/chest.09-1710

[3] Yoshizawa T Okada K Furuichi S Prevalence of chronic kidney diseases in patients with chronic obstructive pulmonary disease: assessment based on glomerular filtration rate estimated from creatinine and cystatin C levels. Int J Chron Obstruct Pulmon Dis 2015;6:1283–9. 10.2147/COPD.S80673 PMC4500615 26185434

[4] Divo M Cote C deTorres JP BODE Collaborative Group. Comorbidities and risk of mortality in patients with chronic obstructive pulmonary disease. Am J Respir Crit Care Med 2012;186:155–61. 22561964 10.1164/rccm.201201-0034OC

[5] Vogelmeier CF Criner GJ Martinez FJ Global strategy for the diagnosis, management, and prevention of chronic obstructive lung disease 2017 report: GOLD executive summary. Am J Respir Crit Care Med 2017;195:557–82. 28128970 10.1164/rccm.201701-0218PP

[6] Decramer M Janssens W Chronic obstructive pulmonary disease and comorbidities. Lancet Resp Med 2013;1:73–83. 10.1016/S2213-2600(12)70060-7 24321806

[7] López-Campos JL Tan W Soriano JB Global burden of COPD. Respirology 2016;21:14–23. 26494423 10.1111/resp.12660

[8] Soriano JB Lamprecht B Chronic obstructive pulmonary disease: a worldwide problem. Med Clin North Am 2012;96:671–80. 22793937 10.1016/j.mcna.2012.02.005

[9] Morgan AD Zakeri R Quint JK Defining the relationship between COPD and CVD: what are the implications for clinical practice? Ther Adv Respir Dis 2018;12: 1753465817750524. doi:10.1177/1753465817750524. 10.1177/1753465817750524 PMC5937157 29355081

[10] Trinkmann F Saur J Borggrefe M Cardiovascular comorbidities in chronic obstructive pulmonary disease (COPD)-current considerations for clinical practice. J Clin Med 2019;10:1–4. 10.3390/jcm8010069 PMC6352261 30634565

[11] Nashef SA Roques F Sharples LD EuroSCORE II. Eur J Cardiothorac Surg 2012;4:735–45.

[12] Zhao H Li L Yang G Postoperative outcomes of patients with chronic obstructive pulmonary disease undergoing coronary artery bypass grafting surgery: a meta-analysis. Medicine (Baltimore) 2019;98:e14388 doi:10.1097/MD.0000000000014388. 30732179 10.1097/MD.0000000000014388 PMC6380818

[13] Ho CH Chen YC Chu CC Postoperative complications after coronary artery bypass grafting in patients with chronic obstructive pulmonary disease. Medicine (Baltimore) 2016;95:e2926 doi:10.1097/MD.0000000000002926. 26937939 10.1097/MD.0000000000002926 PMC4779036

[14] Efird JT Griffin W O’Neal WT Long-term survival after cardiac surgery in patients with chronic obstructive pulmonary disease. Am J Crit Care 2016;25:266–76. 27134234 10.4037/ajcc2016119

[15] Woods SE Bolden T Engel A The influence of chronic obstructive pulmonary disease in patients undergoing coronary artery bypass graft surgery. Int J Med Sci 2010;2:308–13.

[16] Saleh HZ Mohan K Shaw M Impact of chronic obstructive pulmonary disease severity on surgical outcomes in patients undergoing non-emergent coronary artery bypass grafting. Eur J Cardiothorac Surg 2012;42:108–13. 22290913 10.1093/ejcts/ezr271

[17] McKeon NJ Timmins SN Stewart H Diagnosis of COPD before cardiac surgery. Eur Respir J 2015;46:1498–500. 26293502 10.1183/13993003.02339-2014

[18] Cani KC Bonorino KC Gulart AA Pulmonary complications after coronary artery bypass surgery: factors associated factors. ASSOBRAFIR Ciência 2017;8:41–50.

[19] Huang X Redfors B Chen S Impact of chronic obstructive pulmonary disease on prognosis after percutaneous coronary intervention and bypass surgery for left main coronary artery disease: an analysis from the EXCEL trial. Eur J Cardiothorac Surg 2019;55:1144–51. 30596978 10.1093/ejcts/ezy438

[20] Lin WC Chen CW Lu CL The association between recent hospitalized COPD exacerbations and adverse outcomes after percutaneous coronary intervention: a nationwide cohort study. Int J Chron Obstruct Pulmon Dis 2019;14:169–79. 30655664 10.2147/COPD.S187345 PMC6322514

[21] Zhang M Cheng YJ Zheng W Impact of chronic obstructive pulmonary disease on long-term outcome in patients with coronary artery disease undergoing percutaneous coronary intervention. Biomed Res Int 2016;2016:8212459 doi:10.1155/2016/8212459. 28042573 10.1155/2016/8212459 PMC5155073

[22] Ried M Unger P Puehler T Mild-to-moderate COPD as a risk factor for increased 30-day mortality in cardiac surgery. Thorac Cardiovasc Surg 2010;58:387–91. 20922620 10.1055/s-0030-1249830

[23] Shahian DM O’Brien SM Filardo G The Society of Thoracic Surgeons 2008 cardiac surgery risk models: part 1-coronary artery bypass grafting surgery. Ann Thorac Surg 2009;88:S2–2. 19559822 10.1016/j.athoracsur.2009.05.053

[24] Nashef SA Roques F Michel P European system for cardiac operative risk evaluation (EuroSCORE). Eur J Cardiothorac Surg 1999;16:9–13. 10456395 10.1016/s1010-7940(99)00134-7

[25] Nashef SA Roques F Sharples LD EuroSCORE II. Eur J Cardiothorac Surg 2012;41:734–44. 22378855 10.1093/ejcts/ezs043

[26] Shroyer AL Coombs LP Peterson ED The Society of Thoracic Surgeons: 30-day operative mortality and morbidity risk models. Ann Thorac Surg 2003;75:1856–64. 12822628 10.1016/s0003-4975(03)00179-6

[27] O’Boyle Mediratta N Chalmers J Long-term survival of patients with pulmonary disease undergoing coronary artery by-pass grafting. Eur J Cardiothorac Surg 2013;43:697–703. 23096454 10.1093/ejcts/ezs454

[28] Angouras DC Anagnostopoulos CE Chamogeorgakis TP Postoperative and long-term outcome of patients with chronic obstructive pulmonary disease undergoing coronary artery bypass grafting. Ann Thorac Surg 2010;89:1112–8. 20338316 10.1016/j.athoracsur.2010.01.009

[29] Chen CY Liao KM Chronic obstructive pulmonary disease is associated with risk of chronic kidney disease: a nationwide case-cohort study. Sci Rep 2016;6:25855 Published 2016 May 11. doi:10.1038/srep25855. 27166152 10.1038/srep25855 PMC4863146

[30] Kotfis K Strzelbicka M Zegan-Barańska M Validation of the behavioral pain scale to assess pain intensity in adult, intubated postcardiac surgery patients: a cohort observational study - POL-BPS. Medicine (Baltimore) 2018;97:e12443 doi:10.1097/MD.0000000000012443. 30235728 10.1097/MD.0000000000012443 PMC6160138

[31] Kotfis K Zegan-Barańska M Strzelbicka M Validation of the polish version of the critical care pain observation tool (CPOT) to assess pain intensity in adult, intubated intensive care unit patients: the POL-CPOT study. Arch Med Sci 2018;14:880–9. 30002708 10.5114/aoms.2017.69752 PMC6040120

[32] Kotfis K Marra A Ely EW ICU delirium - a diagnostic and therapeutic challenge in the intensive care unit. Anaesthesiol Intensive Ther 2018;50:160–7. 29882581 10.5603/AIT.a2018.0011

